# Analysis of 4999 Online Physician Ratings Indicates That Most Patients Give Physicians a Favorable Rating

**DOI:** 10.2196/jmir.1960

**Published:** 2011-11-16

**Authors:** Bassam Kadry, Larry F Chu, Bayan Kadry, Danya Gammas, Alex Macario

**Affiliations:** ^1^Department of AnesthesiaSchool of MedicineStanford UniversityStanford, CAUnited States; ^2^Eugene Applebaum College of PharmacyWayne State UniversityDetroit, MIUnited States; ^3^School of PharmacyCreighton UniversityOmaha, NEUnited States

**Keywords:** Doctor ratings, patient satisfaction, online physician reviews, consumer health, physician rating

## Abstract

**Background:**

Many online physician-rating sites provide patients with information about physicians and allow patients to rate physicians. Understanding what information is available is important given that patients may use this information to choose a physician.

**Objectives:**

The goals of this study were to (1) determine the most frequently visited physician-rating websites with user-generated content, (2) evaluate the available information on these websites, and (3) analyze 4999 individual online ratings of physicians.

**Methods:**

On October 1, 2010, using Google Trends we identified the 10 most frequently visited online physician-rating sites with user-generated content. We then studied each site to evaluate the available information (eg, board certification, years in practice), the types of rating scales (eg, 1–5, 1–4, 1–100), and dimensions of care (eg, recommend to a friend, waiting room time) used to rate physicians. We analyzed data from 4999 selected physician ratings without identifiers to assess how physicians are rated online.

**Results:**

The 10 most commonly visited websites with user-generated content were HealthGrades.com, Vitals.com, Yelp.com, YP.com, RevolutionHealth.com, RateMD.com, Angieslist.com, Checkbook.org, Kudzu.com, and ZocDoc.com. A total of 35 different dimensions of care were rated by patients in the websites, with a median of 4.5 (mean 4.9, SD 2.8, range 1–9) questions per site. Depending on the scale used for each physician-rating website, the average rating was 77 out of 100 for sites using a 100-point scale (SD 11, median 76, range 33–100), 3.84 out of 5 (77%) for sites using a 5-point scale (SD 0.98, median 4, range 1–5), and 3.1 out of 4 (78%) for sites using a 4-point scale (SD 0.72, median 3, range 1–4). The percentage of reviews rated ≥75 on a 100-point scale was 61.5% (246/400), ≥4 on a 5-point scale was 57.74% (2078/3599), and ≥3 on a 4-point scale was 74.0% (740/1000). The patient’s single overall rating of the physician correlated with the other dimensions of care that were rated by patients for the same physician (Pearson correlation, *r* = .73, *P* < .001).

**Conclusions:**

Most patients give physicians a favorable rating on online physician-rating sites. A single overall rating to evaluate physicians may be sufficient to assess a patient’s opinion of the physician. The optimal content and rating method that is useful to patients when visiting online physician-rating sites deserves further study. Conducting a qualitative analysis to compare the quantitative ratings would help validate the rating instruments used to evaluate physicians.

## Introduction

In 2010, 88% of adult Americans used the Internet to search for health-related information [1-3]. Patients are seeking information not only about disease conditions but also about physicians and hospitals. In fact, in the United States, 47% looked up information about their providers online, 37% consulted physician-rating sites, and 7% of people who sought information about their provider posted a review online [4]. A separate study found that 15% of consumers compare hospitals before making a selection, and 30% of consumers compare physicians online before making a selection [5].

Many physician-rating websites provide users with basic information about the physician such as years in practice and contact information [6,7]. Some of the websites access various databases to display further information about board certification, residency, and any disciplinary action [8]. This information can be obtained for free, or patients can pay to obtain a more in-depth report about the physician [9].

Many websites enable users to enter reviews and rankings about specific physicians. This capability has drawn the attention of consumer advocacy groups, providers, insurance companies, and hospitals. Although knowledge about the patient experience is useful, critics of these portals identify them as being at risk for misinformation, sabotage, and manipulation [10-14]. Few large-scale studies have been conducted to assess the content and rating methods of these physician-rating sites [15].

The goals of this study were to (1) determine the most frequently visited physician-rating websites that have user-generated content, (2) evaluate the content characteristics of each site to rate physicians, and (3) analyze online ratings of 4999 individual physician ratings.

## Methods

Approval for this study was obtained from the Institutional Review Board at Stanford University School of Medicine.

### The Most Commonly Visited Physician-Rating Sites

A search of the Internet (Bing, Google, Google Directory, Google Trends, Blekko, Yahoo, and Yahoo Directory) with search terms *doctor rating*, *physician rating*, *physician-* *rating*, *physician ranking*, and *quality physicians* produced a list of physician-rating sites currently available in the United States [7,15]. On October 1, 2010, using Google Trends, we identified the most commonly visited physician-rating websites using the number of daily unique visits each website attracted [16,17]. Sites with fewer than 5000 daily unique visits as measured on Google Trends were not included in the analyses. Of note, Google Trends is not an absolute measure of Web traffic. The assumption was that the relative Web traffic volume relationship between different websites was consistent. Websites that had Web traffic that registered on Google Trends but did not allow for user-generated content were not included in the analyses. User-generated content was defined as the ability to rate or comment on the physician.

### Rating Content Characteristics of Each Website

We then studied each site to determine the types of rating scales (eg, 1–5, 1–4, 1–100) used and dimensions of care rated (eg, recommend to a friend, waiting room time). All the dimensions of care were identified for each website. To compare different websites, we created a semantic normalization tool. A semantic conversion table was created by first identifying all the different dimensions of care used on each website (Table 1). To facilitate the analysis, each dimension was assigned to 5 categories by three individuals working independently. The 5 different categories were chosen based on the most prevalent rating categories present across various rating websites. There was agreement on 31 of the 35 items, and the group discussed the remaining 4 with the lead author until consensus was reached on the most appropriate category designation: *overall rating, communication skills, access, facilities, and staff*.

**Table 1:** Semantic conversion table used to normalize different dimensions of care used to rate physicians on the websites

Overall rating	Communication Skills	Access	Facilities	Staff
Overall	Communication	Appointments	Office cleanliness	Courteous staff
Level of trust	Explanation	Approachable	Office setting	Staff
Overall quality of care	Explanation of medications	Doctor availability	Office environment	Staff friendliness
Recommendation	Follow-up	Convenience	Service	Staff helpfulness
Recommend to a friend	Attentive during visit	Ease of appointment	Waiting room	Staff professionalism
Patient satisfaction	Listens and answers questions	Quality of referrals	Facilities	Office friendliness
Likely to recommend	Bedside manner	Make Referrals		
	Helps patient understand	Punctuality		

### Analysis of Individual Physician Ratings

Raw data without specific physician identifiers were obtained in October, November, and December 2010 via a nonrandom selection of 4999 online physician ratings from 23 multiple specialties (allergy, cardiology, cardiothoracic surgery, dermatology, endocrinology, gastroenterology, general surgery, hematology, internal medicine, nephrology, neurology, neurosurgery, obstetrics and gynecology, oncology, ophthalmology, orthopedic surgery, otolaryngology, pediatrics, plastic surgery, primary care, pulmonary medicine, rheumatology, and urology) in 25 metropolitan areas (Atlanta, GA; Austin, TX; Baltimore, MD; Boston, MA; Charlotte, NC; Chicago, IL; Colorado Springs, CO; Columbus, OH; Denver, CO; Houston, TX; Los Angeles, CA; Miami, FL; Minneapolis, MN; New Orleans, LA; New York City, NY; Orlando, FL; Phoenix, AZ; Portland, OR; Salt Lake City, UT; San Diego, CA; San Francisco, CA; Raleigh, NC; San Jose, CA; Seattle, WA; and Washington, DC). We chose these cities because they have the highest Internet usage and largest population in the United States [18-20]. The selection of physicians was nonrandom to avoid counting the same physician more than once.

The number of reviews collected from each website varied proportionally by how frequently the websites were visited based on Web traffic estimates from Google Trends. Therefore, the number of reviews from each website was proportional to Web traffic volume assuming that search patterns on Google are similar to those on other search engines.

The sequence of steps followed to acquire each physician rating was to visit the website, enter the city, choose a specialty, enter the largest search radius, and then sort physicians by name when possible. If sorting by name was not possible then location was used. Only reviews that had at least one physician rating completed by a patient within the years 2000–2010 were included in the analyses. Each analyst was assigned a set of metropolitan areas to evaluate physician data.

Cut-offs of 75 (100-point scale), 4 (5-point scale), and 3 (4-point scale) were used to define the favorable threshold for each category of physician-rating website. To compare rankings from different websites with the same rating system, we used a weighted average to accurately represent the overall compiled rating. Only physician-rating sites with the same rating system were compared with one another.

To facilitate analyses, similar dimensions of care—but with different terms used by each website—were grouped into 1 of the 5 categories defined above (*overall rating, access, communication skills, facility, and staff*). For example, wait time, waiting room time, waiting time, and punctuality were all grouped as part of *access* (Table 1).

## Results

### The Most Commonly Visited Physician-Rating Sites

The 10 most commonly visited online physician-rating websites with user-generated content per Google Trends were HealthGrades.com, Vitals.com, Yelp.com, YP.com, RevolutionHealth.com, RateMD.com, Angieslist.com, Checkbook.org, Kudzu.com, and ZocDoc.com (Table 2).

**Table 2:** Top 10 most frequently visited physician-rating websites as a relative measure of Web traffic as measured through Google Trends (October-December 2010)

Website	Percentage	Daily unique visits (per Google Trends)
HealthGrades	40%	254,600
Vitals	20%	127,300
Yelp	15%	95,475
Checkbook	7%	44,555
YP	5%	31,825
ZocDoc	4.8%	30,552
AngiesList	3.2%	20,368
RateMD	3%	19,095
RevolutionHealth	1%	6365
Kudzu	1%	6365
Total	100%	636,500

### Content Characteristics of Each Website

Patients rated 35 different dimensions of care in the websites, with a median of 4.5 (mean 4.9, SD 2.8, range 1–9) dimensions of care per website (Table 1). There was a varying degree of information available on each physician-rating website. Some websites provide users with information on board certification. Some websites have advertisements and other websites provide users the ability to compare physicians side-by-side. Table 3 summarizes information, features, and the presence of advertisements on physician-rating websites.

**Table 3:** Information available on the top 10 physician-rating sites

Website	Comments	Board certification	Years in practice	Physician comparison	Advertising	Sanctions
RateMD	Yes	No	Yes	No	Yes	No
Vitals	Yes	Yes	Yes	Yes	Yes	Yes
AngiesList	Yes	No	Yes	No	No	No
HealthGrades	No	Yes	Yes	No	Yes	Yes
YP	Yes	No	No	No	Yes	No
Kudzu	Yes	No	No	No	Yes	No
Yelp	Yes	No	No	No	Yes	No
ZocDoc	Yes	Yes	No	No	No	No
CheckBook	No	Yes	Yes	Yes	No	No
RevolutionHealth	Yes	Yes	Yes	No	Yes	No

### Analysis of Individual Physician Ratings

The average rating was 77 (308/400, 77.0%) for sites using a 100-point scale (SD 11, median 76, range 33–100). For sites using a 5-point scale the average rating was 3.84 (76.8%, 2764/3599, SD 0.98, median 4, range 1–5). For sites using a 4-point scale the average was 3.1 (77.5%, 774/1000, SD 0.72, median 3, range 1–4).

**Figure 1:**
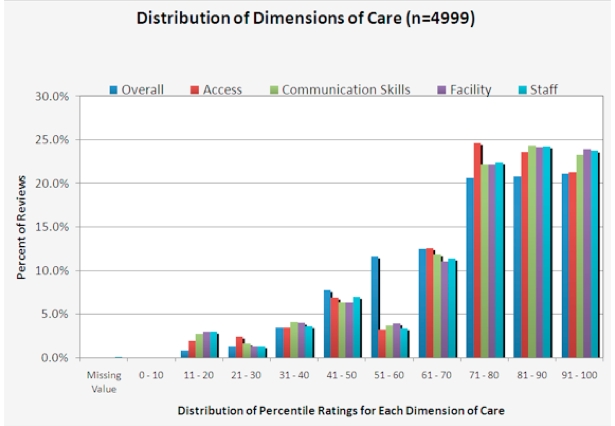
Distribution of percentile ratings for each dimension of care rated on all physician-review sites.

The percentage of reviews with a rating of 75 or higher on physician-rating sites with a 100-point scale was 61.5% (246/400). The percentage of reviews with a rating of 4 or higher on sites with a 5-point scale were 57.74% (2078/3599). The percentage of reviews with a rating of 3 or higher on sites with a 4-point scale were 74.0% (740/100) (Table 4 and Figure 1).

**Table 4:** Physician ratings from the top 10 physician-rating websites with user-generated content. Percentage favorable ratings defined as ≥3 of 4, ≥4 of 5, or ≥75 of 100

Website		Number of reviews evaluated	Percentage of total	Favorable reviews		Overall rating			Lowest rating	Highest rating
				n	%	Mean	SD	Median		
**100-Point scales**										
	Checkbook.org/PatientCentral	350	7%	217	62	77.59	10.48	76.00	34.00	100.00
	RevolutionHealth	50	1%	29	57	74.24	16.01	76.00	33.00	100.00
	Weighted average	400	8%	246	62	77.17	11.17	76.00	33.00	100.00
**5-Point scales**								
	AngiesList	159	3%	103	65	3.95	0.95	4.00	1.00	5.00
	HealthGrades	2000	40%	1139	57	3.82	0.98	4.00	1.00	5.00
	Kudzu	49	1%	26	53	3.74	0.96	4.00	1.00	5.00
	RateMD	150	3%	87	58	3.84	1.00	4.00	1.00	5.00
	Yelp	750	15%	442	59	3.86	0.97	4.00	1.00	5.00
	YP	250	5%	158	63	3.93	0.92	4.00	1.00	5.00
	ZocDoc	241	5%	123	51	3.77	0.92	4.00	1.00	5.00
	Weighted average	3599	72%	2078	58	3.84	0.98	4.00	1.00	5.00
**4-Point scale**								
	Vitals	1000	20%	740	74	3.10	0.72	3.00	1.00	4.00
Total		4999	100%	3064	61.28					

**Figure 2:**
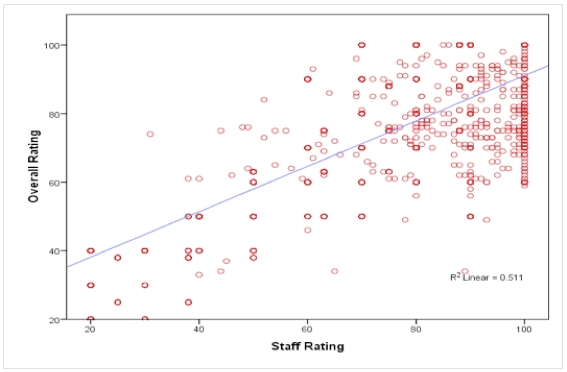
Pearson correlation comparing overall rating versus staff rating (n = 4999, Pearson correlation, *r* = .715, *P* < .001).

The multiple dimensions of care rated by patients on the physician-rating sites with a 5-point scale had a strong correlation with the overall rating (Pearson correlation, *r* = .73, *P* < .001). In fact, the 20 correlations between each of the 5 dimensions of care measured ranged from .715 to .923 (Pearson correlation, *P* < .001). Even the dimension of care with the lowest correlation coefficient with overall rating (ie, staff rating) was significant: Pearson correlation, *r* = .715, *P* < .001) (Figure 2).

## Discussion

### Results are Consistent with Prior Studies

This analysis of 4999 physician ratings across 10 websites revealed that approximately 2 out of 3 patient reviews are favorable. These results are consistent with a study that found that 88% of 190 reviews of 81 Boston physicians were favorable [15]. In that study, a positive rating was defined as a rating of 3 or 4 in sites with a 4-point scale, or 4 or 5 in sites with a 5-point scale. Our results are also consistent with a report that showed that 67% of all Yelp reviews in 2008 were 4 or 5 stars [21,22]. The majority of physician-rating websites depend on subjective data input and offer limited quantitative information about quality and cost of care. Despite these limitations, patients like these websites because they provide insight into the patient experience from peers [23,24]. This issue is becoming more important, as some physicians and hospitals are caught off guard by online reviews that are critical of their services [8-11]. The optimal content, structure, and rating methods for online physician-rating sites that are most useful deserve further study [1,25-27].

### One Feedback Question May be Sufficient to Assess Patient Experience

In all, 35 different dimensions of care were rated by patients in the websites, with an average of 5 questions per site. There was a high correlation between the overall rating of the physician and the other dimensions of care rated (access, communication skills, facility, and staff). This is consistent with using net promoter score methodology to measure customer satisfaction [28]. This raises the issue of whether 1 question may be sufficient to capture the patient’s general experience. In fact, the more questions on a rating site, the less likely a patient will complete the survey [29-32]. A single question such as “Would you recommend Dr X to a loved one?” may be as useful as the multitude of specific questions currently surveyed [33]. Also, from the physician’s point of view, obtaining actionable information to change communication style, facility, or staff may be better obtained by allowing patients to write in specific feedback and commentary rather than by a scaled survey. In other words, if the facility receives a rating of 1 out of 5 stars, and then the patient comments on how dirty the exam rooms were, then the provider will better understand the low rating.

### What makes Physician Ratings Different From Other Professional Service Reviews

Many physicians will take the position that online review sites do not give insight into quality of care. This is valid since obtaining consensus on the definition of quality, even among experts, is challenging. However, patient satisfaction ratings and comments do offer insight into a patient’s experience. As more user-generated content is added, the value of ratings will increase. Patient satisfaction is derived from several factors including the baseline expectation of the patient [25,34,35]. Even government agencies, such as the Consumer Assessment of Healthcare Providers and Systems of the Agency for Healthcare Research and Quality and the value-based purchasing programs proposal introduced by the Center for Medicare & Medicaid Services (CMS), are collecting data on the patient experience [36,37]. CMS even launched a portal of their own to allow for physician comparisons [38]. In fact, the German Medical Association assigned the Agency for Quality in Medicine with the task of elaborating quality standards for online physician- and hospital-rating sites [39]. They suggest that a good online rating site defines how the website is financed, separates rating content from advertising, requires user authentication, provides contact information for the site owner, and allows providers to counter offending statements or correct misinformation.

Despite the overall favorable rating of physicians by patients, the topic of physician ratings is rather sensitive [3,6,10,14,40-47]. Advocates for transparency favor a platform that enables patients to truthfully review their experiences. Yet, with further investigation, a few of these “reviews” have become an outlet for patients who are dissatisfied for not getting what they want despite receiving appropriate medical care. Even worse, some reviews are believed to be acts of sabotage from competing providers or organizations [48-50]. Some physicians have even gone as far as getting a court order to remove a review only to find out that such an action invites Internet vigilantes who find it essential that censorship not be tolerated. Also, patient privacy laws make it very challenging to defend against online misinformation and defamation [48-50]. What makes this issue different from other service industries is that “customers” may die or suffer despite appropriate medical care.

Physician-rating websites hosted by insurance companies have been questioned because of the conflict of interest that insurance companies have by reporting data that can potentially drive patients to providers that are cheap and not because they are good [8]. Consumer review organizations have tried though courts to get access to claims data to report volume of care to the public [51]. However, the American Medical Association and US Department of Health Services and Human won an appeal to protect privacy of physician information. Some physicians request their patients to sign agreements that prohibit them from writing about them on physician-rating websites [49,52,53].

### Limitations

This study has several limitations. There is an implicit selection bias to websites that depend on the user to actively engage the review site and write a review. In the future, to get more feedback, providers may bundle review requests with online services such as appointments (eg, ZocDoc.com) and social networking sites. This may reduce the selection bias that limits the value of physician ratings. We derived physician-rating site traffic from Google Trends, which is not an absolute measure of total site traffic. Also, the authenticity of the review may be in question [48-50].

## Multimedia Appendix 1

Video of the presentation of Dr Kadry at the Medicine 2.0 Congress at Stanford University, September 18th, 2011  http://www.medicine20congress.com/ocs/index.php/med/med2011/paper/view/539.
